# Welcome to the Journal of Medical Internet Research

**DOI:** 10.2196/jmir.1.1.e5

**Published:** 1999-08-11

**Authors:** Gunther Eysenbach

Welcome to the "Journal of Medical Internet Research" - JMIR, the first international scientific peer-reviewed journal on all aspects of research, information and communication in healthcare using Internet and Intranet-related technologies.

Why does the world need the JMIR? The Internet - and more specifically, the World-Wide-Web - has an impact on many areas of medicine - broadly we can divide them into "clinical information and telemedicine", "medical education and information exchange" and "consumer health informatics":

- First, Internet protocols are used for clinical information and communication. In the future, Internet technology will be the platform for many telemedical applications.
- Second, the Internet revolutionizes the gathering, access and dissemination of non-clinical information in medicine: Bibliographic and factual databases are now world-wide accessible via graphical user interfaces, epidemiological and public health information can be gathered using the Internet, and increasingly the Internet is used for interactive medical education applications.
- Third, the Internet plays an important role for consumer health education, health promotion and teleprevention. (As an aside, it should be emphasized that "health education" on the Internet goes beyond the traditional model of health education, where a medical professional teaches the patient: On the Internet, much "health education" is done "consumer-to-consumer" by means of patient self support groups organizing in cyberspace. These patient-to-patient interchanges are becoming an important part of healthcare and are redefining the traditional model of preventive medicine and health promotion).

All these aspects of "cybermedicine" have implications for consumer empowerment and evidence-based medicine: The Internet (or Intranets) enables health professionals to access clinical data just in time, it allows health professionals to access the evidence on the efficacy of available interventions, and finally, it empowers consumers to actively take part in the decision making process.

Clearly, the medical use of the Internet presents enormous opportunities and challenges. The need for research and rapid publication of the findings is obvious. Research in this area should go beyond mere development and provision of technical solutions; it should also address social and human factors, and evaluate the impact of the Internet on society and health care, and public health.

JMIR wishes to publish papers that help physicians and consumers to maximize the use of the Internet. We invite researchers to evaluate the effectiveness and efficiency of Internet communications in health care. We encourage publishers of Internet health information to apply rigorous research methods (such as randomization of users) to evaluate different methods and determinants of communication effectiveness. We invite researchers to compare the effectiveness of communication and information on the Internet with other (traditional) methods of communication. We call for papers that describe and evaluate the effects of the Internet on the patient-physician relationship and the impact on public health. We invite papers that describe the use of the Internet for evidence-based medicine, for example work that demonstrates the development and dissemination of clinical guidelines using the Internet. We wish to receive papers on ethical and legal problems, as well as cross-border and cross-cultural issues, affecting medicine on the Internet, and papers describing possible solutions to the problem of equity of information access. We would like to receive systematic studies examining the quality of medical information available in various online venues. We encourage thought regarding methods of evaluation, quality assessment and improvement of Internet information. We would like to receive proposals for standards in the field of medical publishing on the Internet, including self-regulation issues, policies and guidelines to provide reliable healthcare information. We encourage researchers to experiment with online questionnaires and other data collection experiments such as medical surveys and psychological tests. We would like to publish innovative approaches to use the Internet for healthcare research, examples might include clinical studies, drug reaction reporting and surveillance systems. We would like to publish comments and papers on electronic medical publishing and the use of the Internet for traditional scholarly publishing. We welcome descriptions of websites with innovative content or form, but authors should always make attempts to evaluate the impact of their work, for example trying to determine basic information about user demographics and traffic, where appropriate.

As publishers of a journal *about* the Internet, we are also dedicated to using and experimenting with the Internet as a medium itself. Obviously, we are utilizing the Internet for communication with authors (which is done exclusively by email), and communication with external reviewers, but we also intend to experiment with some novel methods of peer-review. We further invite authors to experiment with innovative methods to communicate their findings, for example submitting HypER-papers (Hypertext Enriched Research Papers) [1] or by the inclusion of animated figures (animated gifs), audio and video into their documents, or by attaching original data which could be downloaded and possibly dynamically analyzed using JAVA applets.

New Internet standards and tools are developing at a breathtaking pace, and many Internet trends have a half-life of less than 6 months. We think that traditional paper journals are simply too slow for the fast-moving field of Internet-technologies. One of our editors reported that an article submitted to a leading medical informatics journal was only published 2 years later - obviously an unacceptable (and unnecessary) delay. Thus, we are trying to publish fast - usually our peer-review time is 1-2 weeks, and we publish all e-papers as soon as they have been accepted (though the printed version may be published later). We will follow a dual publishing strategy - full articles will be published on the Internet, all abstracts and short important articles will be published as a printed version, mainly for archiving and indexing purposes. Our peer-review process will be rigorous and constructive, helping authors to improve their manuscripts and guaranteeing a high-quality journal. We eagerly look forward to your contributions.

Gunther Eysenbach MD

*Editor,*

*Journal of Medical Internet Research*
